# Improved Prediction of Surgical Resectability in Patients with Glioblastoma using an Artificial Neural Network

**DOI:** 10.1038/s41598-020-62160-2

**Published:** 2020-03-20

**Authors:** Adam P. Marcus, Hani J. Marcus, Sophie J. Camp, Dipankar Nandi, Neil Kitchen, Lewis Thorne

**Affiliations:** 1 Queen Elizabeth Hospital, Lewisham and Greenwich NHS Trust, London, UK; 2 Wellcome EPSRC centre for Interventional and Surgical Sciences, University College London, London, UK; 3 Department of Neurosurgery, National Hospital for Neurology and Neurosurgery, UCLH Foundation Trust, London, UK; 4 Department of Neurosurgery, Charing Cross Hospital, Imperial College Healthcare NHS Trust, London, UK

**Keywords:** CNS cancer, Surgical oncology

## Abstract

In managing a patient with glioblastoma (GBM), a surgeon must carefully consider whether sufficient tumour can be removed so that the patient can enjoy the benefits of decompression and cytoreduction, without impacting on the patient’s neurological status. In a previous study we identified the five most important anatomical features on a pre-operative MRI that are predictive of surgical resectability and used them to develop a simple, objective, and reproducible grading system. The objective of this study was to apply an artificial neural network (ANN) to improve the prediction of surgical resectability in patients with GBM. Prospectively maintained databases were searched to identify adult patients with supratentorial GBM that underwent craniotomy and resection. Performance of the ANN was evaluated against logistic regression and the standard grading system by analysing their Receiver Operator Characteristic (ROC) curves; Area Under Curve (AUC) and accuracy were calculated and compared using Wilcoxon signed rank test with a value of p < 0.05 considered statistically significant. In all, 135 patients were included, of which 33 (24.4%) were found to have complete excision of all contrast-enhancing tumour. The AUC and accuracy were significantly greater using the ANN compared to the standard grading system (0.87 vs. 0.79 and 83% vs. 80% respectively; p < 0.01 in both cases). In conclusion, an ANN allows for the improved prediction of surgical resectability in patients with GBM.

## Introduction

Surgical decision-making in patients with glioblastoma (GBM) remains controversial, with little high-quality evidence to guide management. Among the most important of these decisions is whether to perform a biopsy or resection. In each case, a surgeon must carefully consider whether a patient’s tumour is resectable and, in particular, whether sufficient tumour can be removed so that the patient can enjoy the benefits of decompression and cytoreduction, without negatively impacting on a patient’s neurological status.

Defining the surgical resectability of GBM is inherently challenging. As early as 1928 Walter Dandy demonstrated that tumour cells infiltrate far beyond the clinically evident tumour mass^1^. Nonetheless, complete resection of all contrast-enhancing tumour does appear to be associated with significantly improved survival and a multitude of surgical innovations have been introduced to maximise the resection of GBM including fluorescence-guided surgery, and various other intraoperative imaging techniques^2–4^.

The desire for complete resection of all contrast-enhancing tumour must be balanced against the risk of neurological deficits. Surgical resection of GBM continues to carry a significant risk of complications, with new neurological deficits occurring post-operatively in approximately one in ten patients^5,6^. The consequences of these deficits can be severe, affecting quality of life and, ultimately, survival itself.

In a previous study we performed a systematic review of the literature to identify the five most frequently cited anatomical features on a standard pre-operative contrast-enhanced T1-weighted MRI that are predictive of surgical resectability. We then used these features to develop a simple, objective, and reproducible grading system (Table 1)^7^. These features were: periventricular location if the contrast-enhancing tumour was located within 10 mm of the ventricles; bilateral location if the contrast-enhancing tumour extended into the corpus callosum; eloquent location if the contrast-enhancing tumour extended into motor or sensory cortex, language cortex, insula, or basal ganglia; large size if the diameter of the contrast-enhancing tumour exceeded 40 mm; and associated oedema if hypointensity extended more than 10 mm from the contrast-enhancing tumour^7^. All features were weighted equally, with one point assigned if a feature was present, and no points if absent. The sum of these features was then used to describe lesions as low (0–1 points), moderate (2–3 points), and high complexity (4–5 points). The rate of complete of contrast-enhancing tumour varied widely from 3.4% in high complexity lesions to 50.0% in low complexity lesions^7^.

**Table 1:** Previously reported grading system for adults with supratentorial glioblastoma. All features are assessed using the pre-operative contrast-enhanced T1-weighted MRI^7^.

Pre-operative MRI feature	Score
Periventricular or deep location	
≥10 mm from ventricle	0
<10 mm from ventricle	1
Corpus callosum or bilateral location	
No corpus callosum involvement	0
Corpus callosum involvement or bilateral location	1
Eloquent location	
Not eloquent location	0
Eloquent location (motor or sensory cortex, language cortex, insula or basal ganglia)	1
Largest diameter of tumour	
<40 mm	0
≥40 mm	1
Associated oedema	
<10 mm from contrast-enhancing tumour	0
≥10 mm from contrast-enhancing tumour	1
**TOTAL**	**0–5**
	0–1 Low complexity
	2–3 Moderate complexity
	4–5 High complexity

Machine learning is a branch of artificial intelligence that may improve outcome prediction beyond standard grading systems by accounting for the more complex relationships between inter-related variables. While previous studies have demonstrated the utility of machine learning within neurosurgery, its use generally requires very large datasets limiting its application^8^. To this end, a novel framework has been recently been reported that allows for the application of artificial neural networks (ANN), a type of machine learning, to small datasets^9^. In this study we apply a variation of this framework to predict surgical resectability in patients with GBM.

## Materials and Methods

Ethical approval to develop and validate a resectability grading system using a prospectively maintained database was obtained from our local Research Ethics and Audit Committees. Informed consent was not deemed necessary by these Committees, as a retrospective study design was used. All methods were performed in accordance with the relevant local guidelines and regulations.

### Dataset

A detailed description of the methods used to acquire the original dataset has previously been reported^7^. In brief, a prospectively maintained database was searched between the 1^st^ January 2014 and the 31^st^ June 2015 to identify all adult patients with supratentorial GBM that underwent craniotomy and resection at a University Teaching Hospital. In each patient, the latest pre-operative contrast-enhanced T1-weighted MRI scan (usually within 72 hours before surgery) was scored by two neurosurgeons blinded to the outcome using the aforementioned grading system (Table 1). The earliest post-operative contrast-enhanced T1-weighted MRI scans (usually within 72 hours after surgery) was then evaluated by a consultant neuroradiologist blinded to the grade to determine the extent of resection (complete resection of all contrast-enhancing tumour, or not). A retrospective case note review was also performed to identify any immediate surgical complications, which were recorded according the Clavian-Dindo classification^10,11^.

Our new dataset was obtained by applying the same methodology to patients operated on between the 1^st^ February 2017 and the 1^st^ August 2017 at another University Teaching Hospital. This was combined with our original dataset and used to develop and validate the ANN model.

All operations were performed by six specialist neurosurgeons, who spend at least half of their clinical programmed activity in neuro-oncology. Intraoperative imaging including 5-Aminolevulinic Acid (5-ALA), ultrasound, and MRI were used according to availability and surgeon preference. However, only the pre- and post-operative MRI data were used to develop and validate the ANN model.

### Network design

Artificial neural networks, like biological neural networks, consist of several neurones that are connected together. In this study, a multilayer perceptron network was used that consisted of at least three layers arranged in a series: an input layer, a number of hidden layers, and an output layer. The input layer contained five neurones corresponding to each of the five important anatomical features identified on pre-operative MRI. The output layer contained a single neurone representing the probability of complete surgical resection of the contrast-enhancing tumour. Neurones were connected using a feed-forward structure that allowed signals to travel from input to output only. Neurones within the input layer passed their output to the first hidden layer, neurones in this layer then pass their output to the second hidden layer, until eventually the output layer was reached.

An ANN consists of a number of parameters. The majority of these are learned through training however several of these need to be set before the learning process begins and are termed hyperparameters. Our network defined the following hyperparameters: training algorithm, training algorithm parameters, learning rate, learning momentum, number of hidden layers, number of hidden neurones per hidden layer, neurone activation functions, weight initialisation method, and weight initialisation parameters.

### Network selection

An evolutionary approach was used to optimise the network hyperparameters. An initial population of 100 solutions were generated with random tuples of these hyperparameters. The performance of each solution was evaluated using 100 repeats of 10-fold stratified cross-validation. In this procedure the dataset is randomly partitioned into 10 equal sized subsamples while ensuring approximately proportional contributions of patients with complete resection of all contrast-enhancing tumour, or not. Each subsample was used once as validation data for testing the model while the other samples were used as training data. This was repeated 100 times to mitigate high variance issues arising from small data conditions^9^. The results were then averaged to produce a single estimation of performance.

The Fast Artificial Neural Network (FANN) library version 2.2.0 was used to implement the ANN’s. The cost function used during training was chosen from this library, and defined by the mean squared error (MSE) between the output and actual value representing if the contrast-enhancing tumour was completely resected. Early stopping was implemented to avoid overfitting and increase generalisation^12^. The fitness of each solution was calculated using the average validation error of the set of ANN’s. For each generation the 90 worst performing solutions were replaced with new solutions with hyperparameter tuples generated by crossover and random mutation. Uniform crossover was used where each hyperparameter was selected from parents with equal probability. The rate of random mutation was decayed exponentially from 100% in the first generation to 5% using factor of 0.85. After 100 generations the single best performing solution of hyperparameter tuples was selected.

The final ANN model was an ensemble of 1000 ANN’s. Diversification of the constituent ANN’s was achieved by randomising initial parameters and training on different pairs of training and validation sets. ANN’s outputs were then combined using simple averaging. This strategy has shown to be effect when dealing with small datasets^13,14^. Training the ensemble used the same approach as training the solutions in evolutionary hyperparameter optimisation. The cost function was MSE and early stopping was used to improve performance.

### Network evaluation

For comparison of the ANN, logistic regression, and standard models, averaged receiver operating characteristic (ROC) curves were created using 10 repeats of 10-fold stratified cross-validation (in effect we used nested cross-validation with an outer loop for network evaluation and inner one for network selection)^15^.

A probability cut off point of 0.5 (50%) was applied to the ANN and logistic regression models to classify the predicted resectability on the pre-operative MRI as low complexity (resectable) or high complexity (not resectable). For the standard grading system, low complexity lesions (0–1 points) were considered resectable, and more complex lesions (2–5 points) were considered not resectable; this cut off was chosen because our previous study found that moderate and high complexity lesions had a less than 50% rate of resection. The overall accuracy of the final model was determined by comparing the predicted resectability and whether actual complete excision of all contrast enhancing tumour was achieved as judged on the post-operative MRI. The area under the curve (AUC) from the ROC analysis was evaluated to compare the discriminatory power of the models. Associated goodness-of-fit statistics (specificity, sensitivity, negative and positive predictive values) were calculated with the same approach.

We employed a cross-validated paired Wilcoxon signed rank test to establish the statistical significance of the difference in performance between two models, with a p < 0.05 considered statistically significant. Wilcoxon signed rank test was chosen in place of the more commonly used t-test due to its better tolerance of outliers and improved suitability towards machine learning datasets^16^. All statistical analyses were performed using R version 3.4.4. (R Foundation for Statistical Computing, Vienna, Austria).

## Results

### Dataset

In total, 135 patients were included, of which 33 (24.4%) were found to have complete excision of all contrast-enhancing tumour. The median age was 60 years (interquartile range 47–70 years), and the male:female ratio was 2.1:1. The median length of stay was 5 days (interquartile range 4–11 days). Three patients had major complications (2.2%): two had intracerebral haematoma, and one had pulmonary embolism.

### Network selection

**Figure 1:**
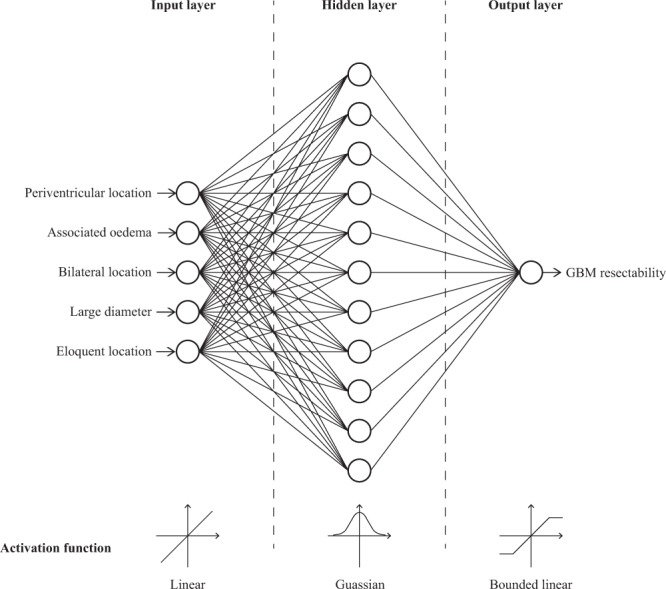
Median Artificial Neural Network (ANN).

The median optimal hyperparameters determined by our evolutionary approach are reported in Table 2, and the median network structure illustrated in Fig. 1. The median network structure had three layers: an input layer containing five neurones corresponding to each of the five important anatomical features identified on pre-operative MRI; a single hidden layer containing 11 neurones; and an output layer representing the probability of complete surgical resection of the contrast-enhancing tumour.

**Table 2:** Median optimal hyperparameter values determined by our evolutionary approach.

Hyperparameter	Median optimal value	Possible values
Number of hidden layers	1	1–100
Number of neurones in hidden layer	11	1–1000
Hidden layer activation function	Gaussian	Linear Bounded linear Sigmoid Gaussian
Hidden layer activation steepness	0.400061	0–1
Output layer activation function	Bounded linear	Linear Bounded linear Sigmoid Gaussian
Output layer activation steepness	0.05962545	0–1
Training algorithm	Resilient Backpropagation (RPROP)	Incremental Resilient Backpropagation (RPROP) Quickprop Simulated Annealing Enhanced Resilient Backpropagation (SARPROP)
Initial step size (Δ_zero_)	0.257795	0–1
Maximum step size (Δ_max_)	226.582	0–500
Minimum step size (Δ_min_)	0.05381755	0–0.1
Decrease factor (η^−^)	0.676261	0–1
Increase factor (η^+^)	1.43495	1–10
Weight initialisation method	Random	Random Widrow + Nguyen’s algorithm
Minimum initial weight	−0.4201585	−1–0
Maximum initial weight	0.1856795	0–1

### Network evaluation

**Figure 2:**
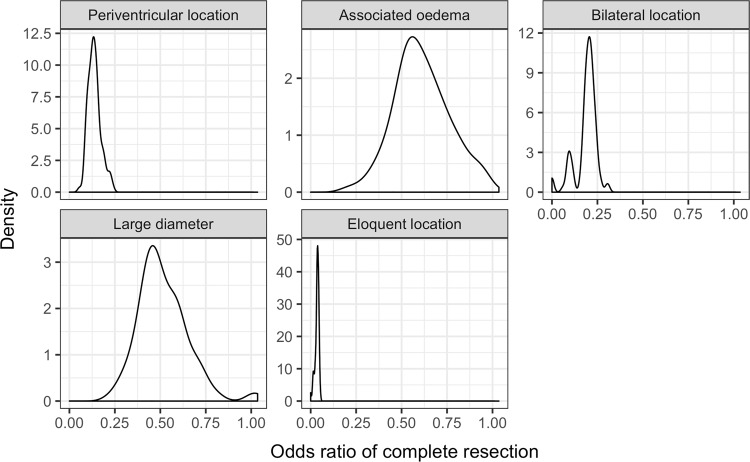
Distribution of logistic regression (LR) coefficients across cross-validation folds. Coefficients represent the odds ratio of presence of pre-operative MRI features compared to no MRI features.

The most important factors identified by logistic regression were: eloquent location, deep location, and bilateral location (Fig. 2).

**Figure 3:**
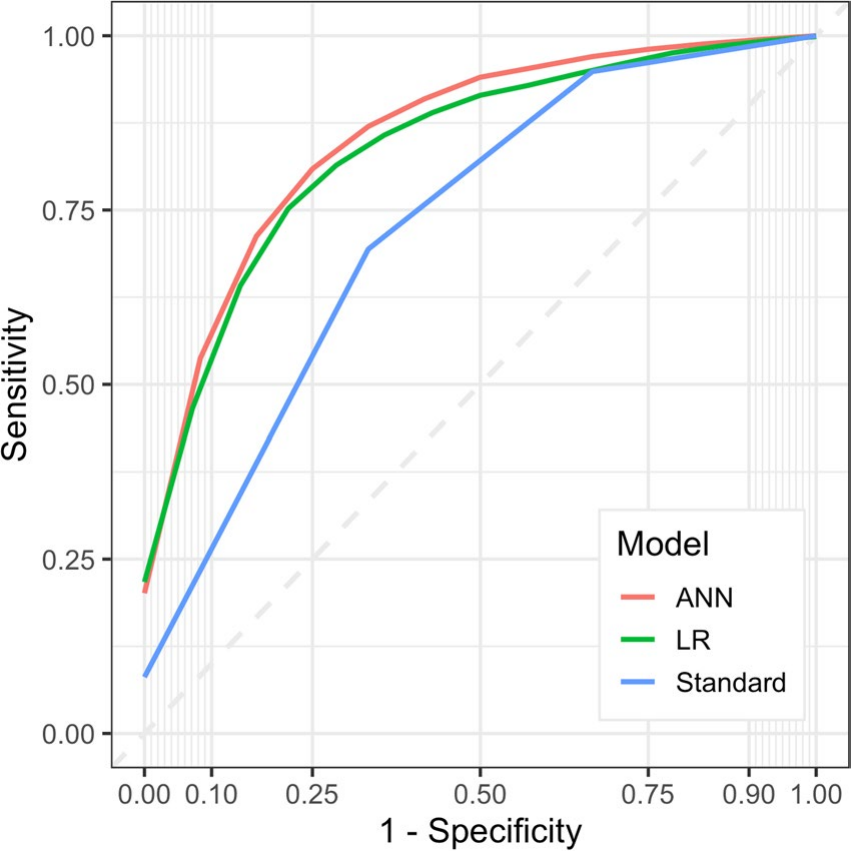
Mean Receiver Operating Characteristic (ROC) curves using the Artificial Neural Network (ANN), logistic regression (LR), and standard grading system to predict surgical resectability in patients with glioblastoma.

The comparative performance of the ANN against logistic regression and the standard grading system is reported in Table 3, and the mean ROC illustrated in Fig. 3. The AUC and accuracy were significantly greater using the ANN compared to the standard grading system (0.87 vs. 0.79 and 83% vs. 80% respectively; p < 0.01 in both cases).

**Table 3:** Mean performance using the standard grading system, logistic regression (LR), and Artificial Neural Network (ANN) to predict surgical resectability in patients with glioblastoma.

Parameter	Estimate (95% CI)	P value
**Accuracy**		
ANN	83.4 (81.6–85.1)	<0.01
LR	83.2 (81.3–85.0)	<0.01
Standard grading system	80.2 (78.2–82.1)	Reference
**AUC**		
ANN	0.871 (0.849–0.895)	<0.01
LR	0.868 (0.848–0.889)	<0.01
Standard grading system	0.786 (0.747–0.825)	Reference
**Sensitivity**		
ANN	0.586 (0.531–0.640)	0.0861
LR	0.587 (0.532–0.643)	0.0807
Standard grading system	0.543 (0.489–0.560)	Reference
**Specificity**		
ANN	0.915 (0.898–0.932)	0.0117
LR	0.910 (0.893–0.927)	0.0380
Standard grading system	0.885 (0.861–0.908)	Reference
**PPV**		
ANN	0.707 (0.649–0.766)	0.0164
LR	0.679 (0.619–0.739)	0.236
Standard grading system	0.642 (0.585–0.698)	Reference
**NPV**		
ANN	0.877 (0.862–0.892)	0.0945
LR	0.878 (0.862–0.893)	0.0677
Standard grading system	0.864 (0.849–0.880)	Reference

**Figure 4:**
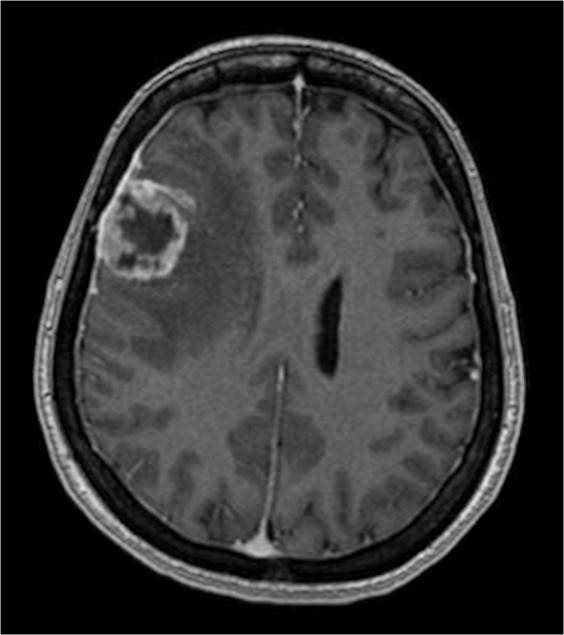
T1-weighted gadolinium-enhanced axial MRI brain demonstrating a low complexity lesion with a 74.9% likelihood of complete resection.

**Figure 5:**
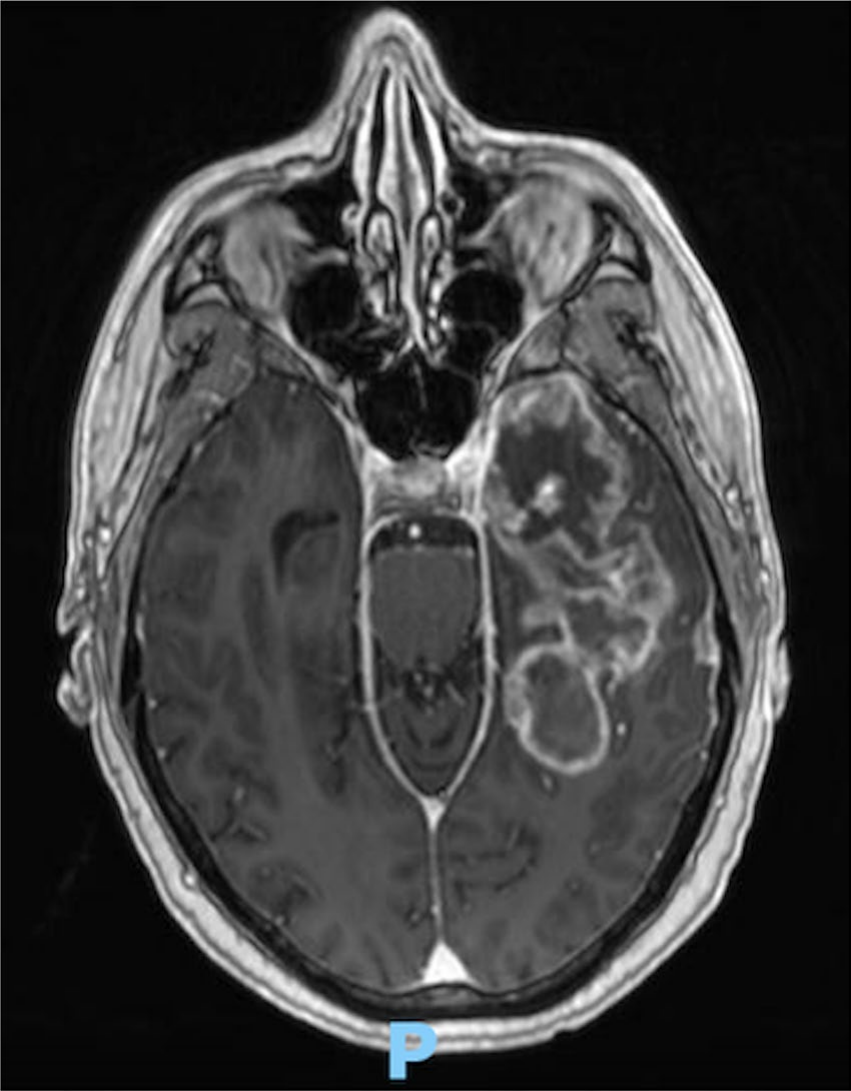
T1-weighted gadolinium-enhanced axial MRI brain demonstrating a high complexity lesion with a 5.7% likelihood of complete resection.

Examples of low and high complexity tumours, as assessed by the ANN, are illustrated in Figs. 4 and 5 respectively.

## Discussion

### Principal findings

Currently, there is no consensus on surgical resectability in patients with GBM, with considerable variation in clinical judgement between surgeons reflecting different surgical philosophies. In a study at the University of Michigan, Orringer *et al*. found that two surgeons were more likely to disagree than agree with each other as to whether the features of a particular GBM are amenable to complete excision^17^. Even when surgeons agreed the features of a GBM allowed for complete excision, this was achieved in less than a quarter of cases.

While individual surgeons vary considerably in their clinical judgement of surgical resectability, pooled responses from of a large number of surgeons has been shown to be far more consistent and predictive. Sonabend *et al*. found that the surgical resectability in patients with GBM calculated from the pooled responses of 13 surgeons was strongly correlated with the percentage of contrast-enhancing tumour^18^.

In a previous study we attempted to address the definition and evaluation of surgical resectability and proposed a simple, objective, and reproducible grading system that allowed for the standardised reporting of the five most important features of GBM on pre-operative MRI. The present study has furthered this work, and developed an ANN to predict surgical resectability. In order to develop the ANN using a comparatively small dataset, we employed several strategies drawn from the literature including: use of an ensemble of ANN’s^14^; evaluation of performance using k-fold cross-validation^14^; and the method of multiple runs, where a large number of models are trained, and performance is averaged^9^.

We have demonstrated that use of the aforementioned ANN does improve the prediction of surgical resectability in patients with GBM. The clinical significance of this improved prediction remains uncertain, particularly in comparison to logistic regression. However, we hope that use of the ANN, which we have made freely available online and as a mobile app (https://amarcu5.github.io/GBM-resectability-prediction/), will aid surgical decision making, and also provide a basis for more robust comparative effectiveness research when reported alongside the surgical outcome of patients undergoing craniotomy for GBM.

### Comparison with other studies

To the best of our knowledge, this is the first study describing the use of an ANN to predict surgical resectability in patients with GBM. However, machine learning has been used elsewhere for neurosurgical outcome prediction in patients with brain tumours and other conditions such as neurovascular disease, epilepsy, movement disorders, traumatic brain injury, and hydrocephalus^8,19,20^. A recent systematic review has found that machine learning models perform significantly better than logistic regression, with a median absolute improvement in the AUC and accuracy of 0.06 and 15% respectively^8^. Moreover, in many cases machine learning models were found to outperform clinical experts, with a median absolute improvement in the AUC and accuracy of 0.14 and 13% respectively^19^.

Previous studies using machine learning predict the outcome in patients with brain tumours have an AUC between 0.76 and 0.85, which compares favourably with our own studies AUC of 0.87^21–25^. In the largest of these studies, Emblem *et al*. used a generic support vector machine model (another form of machine learning) in combination with MR imaging–based whole-tumour relative cerebral blood volume (rCBV) histograms to predict 6-month and 1-, 2-, and 3-year survival, and reported an AUC between 0.79 and 0.85^21^. Akbari *et al*. also used a generic support vector in combination with advanced imaging techniques such as Diffusion Tensor Imaging (DTI) and Dynamic Susceptibility Contrast (DSC) MR perfusion to predict early recurrence, and reported an AUC of 0.84. Among these studies using machine learning predict the outcome in patients with brain tumours, our work is unique in: (1) using an ANN rather than other machine learning models; (2) using standard pre-operative contrast-enhanced T1-weighted MRI rather than advanced imaging techniques; and (3) predicting resectability rather than overall survival or progression free survival.

### Limitations

The present study has several limitations. First, we used quantised data generated using the standard grading system, rather than the raw imaging data, to train and evaluate the ANN. The use of quantised data allowed for a fair comparison of the performance of the ANN against the standard grading system, but we anticipate that performance of the ANN would be further improved using the raw imaging data. Second, we used a small and retrospective study design. The choice of study design was pragmatic but introduced the possibility of bias, and made the accurate and precise coding of post-operative neurological status and complications challenging. In this respect high-quality cognitive assessments are very important to better assess loss of functional capacity in language and executive domains. Third, the low rate of surgical complications identified did not allow for their incorporation into the ANN. More generally, it is recognised that each surgeon will vary in how they balance their desire for complete resection against the risk of neurological deficits. These limitations will largely be addressed in a planned prospective multicentre study.

## Conclusions

The proposed ANN allows for the improved prediction of surgical resectability in patients with GBM. Although the clinical significance of this remains uncertain, it is hoped that use of the ANN in clinical practice and in the literature will serve as a helpful adjunct to surgical decision making, and allow for more meaningful comparison between studies reporting the surgical outcome of patients undergoing craniotomy for GBM. In future work we hope to expand the features of the ANN, reporting the probability of near-complete alongside complete excision of contrast-enhancing tumour, and also the probability of major complications.

### Ethical approval

Ethical approval to develop a resectability grading system using the original dataset was obtained from the Joint Research Compliance Office (JRCO), Imperial College London. Subsequent approval to validate the grading system on a new dataset was by the Audit Committee, National Hospital for Neurology and Neurosurgery.

### Informed consent

Informed consent was not sought as a retrospective study design was used. Images were fully anonymised so as not reveal patient identity.

### Originality

Interim findings of this work were shared as an oral presentation at the meeting of the Society of British Neurological Surgeons in London, September 2018.

### Copyright

The Corresponding Author has the right to grant on behalf of all authors and does grant on behalf of all authors, a worldwide licence to the Publishers and its licensees in perpetuity, in all forms, formats and media (whether known now or created in the future), to (i) publish, reproduce, distribute, display and store the Contribution, (ii) translate the Contribution into other languages, create adaptations, reprints, include within collections and create summaries, extracts and/or, abstracts of the Contribution, (iii) create any other derivative work(s) based on the Contribution, (iv) to exploit all subsidiary rights in the Contribution, (v) the inclusion of electronic links from the Contribution to third party material where-ever it may be located; and, (vi) licence any third party to do any or all of the above.
